# Coulomb focusing in attosecond angular streaking

**DOI:** 10.1038/s41377-024-01600-4

**Published:** 2024-09-11

**Authors:** Xiaokai Li, Xiwang Liu, Chuncheng Wang, Shuai Ben, Shengpeng Zhou, Yizhang Yang, Xiaohong Song, Jing Chen, Weifeng Yang, Dajun Ding

**Affiliations:** 1 Institute of Atomic and Molecular Physics, and Advanced Light Field and Modern Medical Treatment Science and Technology Innovation Center of Jilin Province, Jilin University, Changchun, 130012 China; 2 School of Physics and Optoelectronic Engineering, Hainan University, Haikou, 570228 China; 3 Hefei National Laboratory, and Hefei National Research Center for Physical Sciences at the Microscale and School of Physical Sciences, University of Science and Technology of China, Hefei, 230026 China; 4 Center for Theoretical Physics, Hainan University, Haikou, 570228 China

**Keywords:** Optical physics, Optical metrology

## Abstract

Angular streaking technique employs a close-to-circularly polarized laser pulse to build a mapping between the instant of maximum ionization and the most probable emission angle in the photoelectron momentum distribution, thereby enabling the probe of laser-induced electron dynamics in atoms and molecules with attosecond temporal resolution. Here, through the jointed experimental observations and improved Coulomb-corrected strong-field approximation statistical simulations, we identify that electrons emitted at different initial ionization times converge to the most probable emission angle due to the previously-unexpected Coulomb focusing triggered by the nonadiabatic laser-induced electron tunneling. We reveal that the Coulomb focusing induces the observed nonintuitive energy-dependent trend in the angular streaking measurements on the nonadiabatic tunneling, and that tunneling dynamics under the classically forbidden barrier can leave fingerprints on the resulting signals. Our findings have significant implications for the decoding of the intricate tunneling dynamics with attosecond angular streaking.

## Abstract (web summary)

This study demonstrates the Coulomb focusing in nonadiabatic tunneling ionization inducing the convergence of electrons emitted at different initial ionization times to the same emission angle, thereby breaking the one‐to-one correspondence—one of the fundamental assumption in attosecond angular streaking.

## Graphical abstract

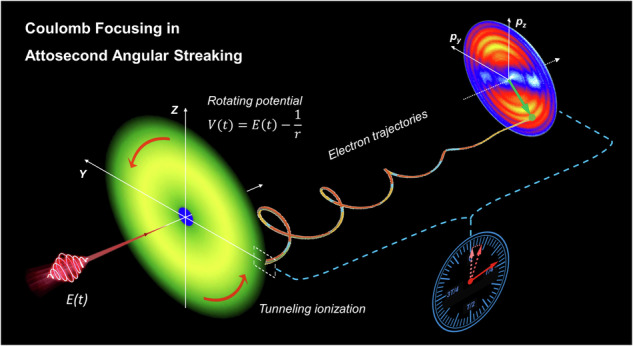

## Introduction

Attosecond metrologies, such as attosecond streaking with isolated attosecond pulses^1^, attosecond photoelectron interferometry with attosecond pulse train^2,3^ and attosecond angular streaking^4–8^, have successfully revealed the temporal nature of photoionization in an unprecedented time scale, which are essential to achieve the quantum control of attosecond chemistry^9^ and the petahertz-scale signal processing at optical frequencies^10,11^. Among those techniques, angular streaking is a sophisticated method for investigating the electron tunneling dynamics with attosecond resolution based on femtosecond laser pulse^4,5,7,12,13^. The rotating electric field vector of the close-to-circularly polarized laser pulse is employed to deflect the direction of electron emission, and the instant of ionization was assumed to map to the most probable emission angle (MPEA) in the polarization plane. With this technique, numerous experimental and theoretical studies have been implemented to reveal the temporal information of electron tunneling, but different conclusions have been drawn regarding the duration of tunneling ionization, with some studies suggesting it takes tens of attoseconds while others proposing it to be instantaneous^5,6,8,13–20^.

Through a comparison of the attoclock measurements on atomic hydrogen and the time-dependent Schrödinger equation (TDSE) simulations with Yukawa potential, Satya et al. concluded that the observed MPEA in the final photoelectron momentum distribution (PMD) is exclusively attributed to the post-tunneling Coulomb interaction^13^. Energy-resolved angular streaking measurements performed on the same target in the nonadiabatic regime reveal an increase in the MPEA with energy, contradicting the standard predictions based on Coulomb interaction with the ion^21^. In order to address the inconsistencies in the measurements, it is necessary to investigate the validity of the widely used assumptions underlying angular streaking. The time-angle mapping relationship in angular streaking technology is built on the assumption that the photoelectrons contributing to MPEA originate from the tunneling burst created at the instant of electric field peak. A one-to-one correspondence between ionization time and emitted direction of electron was supposed to be guaranteed by the near-circularly polarized laser pulse which excludes rescattered electron^4,5,22,23^. But the situation is complicated by the fact that the post-tunneling Coulomb interaction distorts the evolution of the liberated electron, indicating that angular streaking is not capable of directly tracing the initial ionization time from the MPEA in the final PMD after the Coulomb interaction in the continuum.

In this work, we report that in angular streaking measurements with strong near-circularly polarized laser fields, the MPEA is contributed by electrons that penetrate through the barrier at different initial ionization times, owing to the previously-unexpected Coulomb focusing triggered by the nonadiabatic tunneling. By comparing the semiclassical statistical analysis based on an improved Coulomb-corrected strong-field approximation (ICCSFA) (see Supplementary Material) with experimental observations, we demonstrate that the Coulomb focusing induces the counterintuitive energy-dependent trend, i.e., the increase of the MPEA with energy. Moreover, we identify that the sub-barrier Coulomb attraction leads to smaller tunneling exit positions and thus stronger Coulomb focusing in the continuum, which leaves fingerprints on the measured angular streaking signals.

## Results

**Fig. 1:**
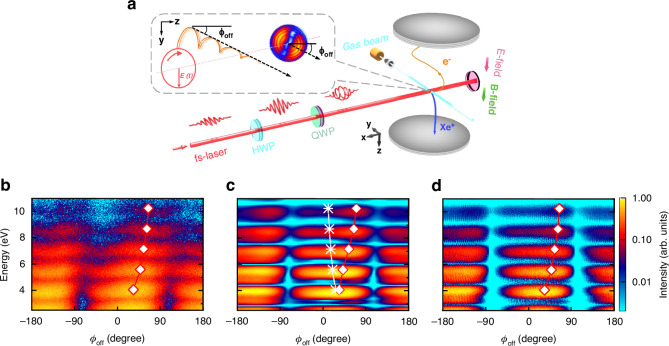
Energy-resolved attosecond angular streaking. **a** Schematic of the experimental measurement with angular streaking. Energy-dependent photoelectron angular distribution acquired from (**b**) measurements; (**c**) numerical solution of TDSE; and (**d**) ICCSFA simulations. The white diamonds denote the measured or simulated MPEA of photoelectrons, and the white stars represent the simulated angle of the final momentum of the electron emitted at the instant of the electric field peak. The peak laser intensity is (45 ± 2) TW cm^−2^ for the measurements and 45 TW cm^−2^ for the simulations. The color bar stands for the normalized intensity of the measured or calculated photoelectron yields in logarithmic scale

**Fig. 2:**
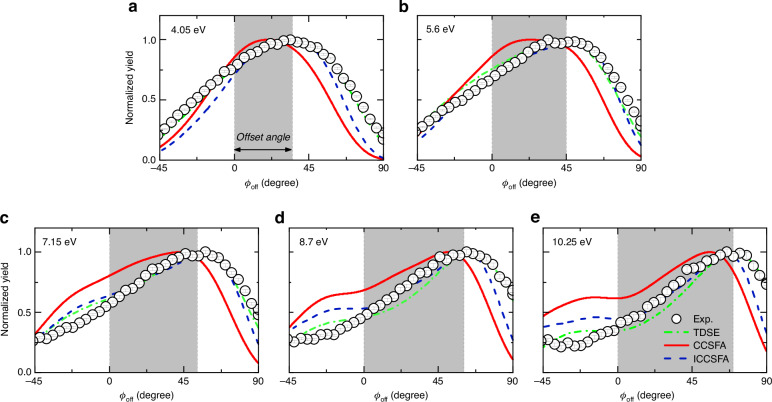
Angular offsets of electrons from different ATI orders. The measured (black dots with errorbar) and calculated (curves) angular distributions for ATI rings with different electron energies of (**a**) 4.05 eV, (**b**) 5.6 eV, (**c**) 7.15 eV, (**d**) 8.7 eV and (**e**) 10.25 eV. The offsets of the MPEAs from the experimental measurement (shaded areas) and those calculated from TDSE and ICCSFA simulations demonstrate a quantitative agreement, which shows clear angle shifts relative to the CCSFA calculations without the sub-barrier potential interaction

Experimentally, we performed energy-resolved angular streaking measurements on various noble gas atoms including Ar, Kr and Xe with Cold-Target Recoil-Ion Momentum Spectroscopy (COLTRIMS)^24–26^ (see Fig. 1a for the experimental scheme). For Xe atom, the employed peak intensity is ~45 TW cm^-2^ and the corresponding Keldysh parameter is ~2, indicating that it is within the typical nonadiabatic tunneling regime^27,28^. Multiple cycle laser pulses with durations of 40 fs are adopted to scale the energy of photoelectron with above-threshold ionization (ATI) rings spaced by the energy of a single photon. The inset in Fig. 1a displays the measured angular streaking electron momentum spectra and schematically illustrates the electron trajectories (depicted as orange lines) under the simultaneous interaction of a rotating electrical field and long-range Coulombic attraction. The *x*, *y* and *z* axes represent the directions of laser propagation, major and minor axes of polarization ellipse of the laser field, respectively. The angular offset ${\phi }_{{\rm{off}}}$ of the photoelectron is defined as the angle between the asymptotic direction of electron and the *z* axis. Figure 1b illustrates the energy-resolved angular distribution from the angular streaking measurement of Xe. The measured MPEAs show an increasing trend with energy, which is also observed for Kr and Ar (see Fig. S1 in Supplementary Material) as well as for hydrogen atom in the literature^21^. This suggests that the energy-dependent trend of MPEAs is a universal phenomenon in angular streaking measurements of atoms. The measured energy-dependent MPEAs are well reproduced by three-dimensional TDSE simulations^29,30^, as shown in Fig. 1c. The one-dimensional distributions of the measurements and TDSE calculations for five ATI rings are presented in Fig. 2a–e, demonstrating the quantitative agreement of MPEAs (the green dash dot lines in Fig. 2a–e).

To provide a transparent interpretation on the measured results of angular streaking, we perform ICCSFA simulations based on Feynman path integral and semiclassical statistical analysis of electron trajectories (see “Methods” and Supplementary Material)^31–37^. Unlike most Coulomb-corrected semiclassical methods, such as Coulomb-corrected strong-field approximation (CCSFA), the ICCSFA method incorporates Coulomb interactions not only in the continuum but also under the classically forbidden tunneling barrier. The ICCSFA has already been successfully employed to reproduce the experimental results of a molecular attoclock and to extract the resonant time delay from the measurements^35^. The energy-dependent trend of MPEAs in the measurement and TDSE simulation is quantitatively reproduced by the ICCSFA simulation (see Fig. 1d and the blue dash lines in Fig. 2a–e). In contrast, when the sub-barrier Coulomb interaction is artificially switched off, the CCSFA simulation fails to reproduce the measured MPEAs (see the solid red lines in Fig. 2a–e).

**Fig. 3:**
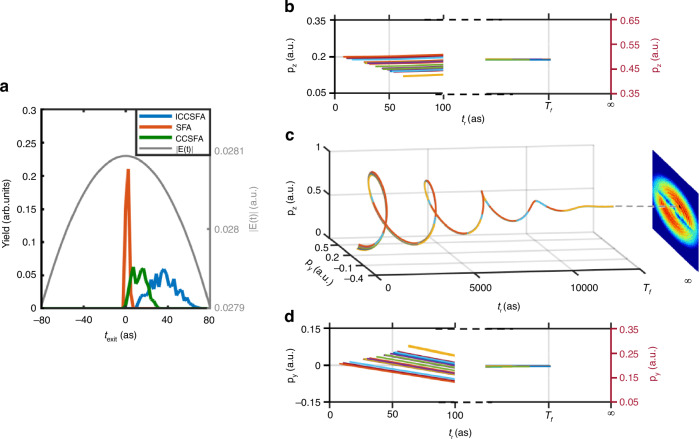
Semiclassical statistical analysis of the initial distribution of electron trajectories. **a** The distribution of initial ionization times for the photoelectron contributing to the MPEA in the first ATI. The instant of the electric field peak is assumed as time zero. **c** Simulated time-dependent evolution of electrons from the momentum bin of MPEA. **b**, **d** The time-dependent evolution of photoelectron momentum projections in the *z* and *y* directions, respectively. *T*_f_ is the moment the laser field ends. The colors of the trajectories are used to guide the eye

Subsequently, we employ the semiclassical statistical analysis to trace back the initial distribution of electron trajectories of the angular streaking signal. Figure 3a depicts the calculated initial time distributions of all electron trajectories contributing to the momentum bin of MPEA of the first ATI ring with three approaches including ICCSFA (blue line), CCSFA (green line) and strong-field approximation (SFA) (orange line). When the Coulomb interaction is artificially switched off in SFA, electrons are released at time 0, i.e., the instant of the electric field peak. This agrees with the simulation within the single classical trajectory (SCT) approximation^19,21^. It was in accord with the original hypotheses of angular streaking that maximum ionization was triggered at the instant of the electric field peak (SFA, orange line in Fig. 3a), which is the base of the one-to-one correspondence between the instant of maximum ionization and the MPEA. As a comparison, when the Coulomb interactions during the tunneling and in the continuum are fully taken into account in ICCSFA simulations, the ionization does not occur exactly at the instant of the electric field peak, but instead spans during a duration more than 60 as (see the blue line in Fig. 3a). Even when only the Coulomb interaction in the continuum is considered (CCSFA), the ionization also occurs during a duration of approximately 30 as (see the green line in Fig. 3a). Moreover, in ICCSFA simulations, the statistical analysis on trajectories contributing to the MPEA shows that numerous electron trajectories beginning at different initial ionization times are converged into the same final momentum by the Coulomb focusing, as illustrated in Fig. 3b–d (also schematically shown in the inset of Fig. 1a), which selects a momentum bin from the MPEA of the first ATI as an example. Therefore, the electrons contributing to the MPEA are not released at a specific moment, but rather over a time span.

**Fig. 4:**
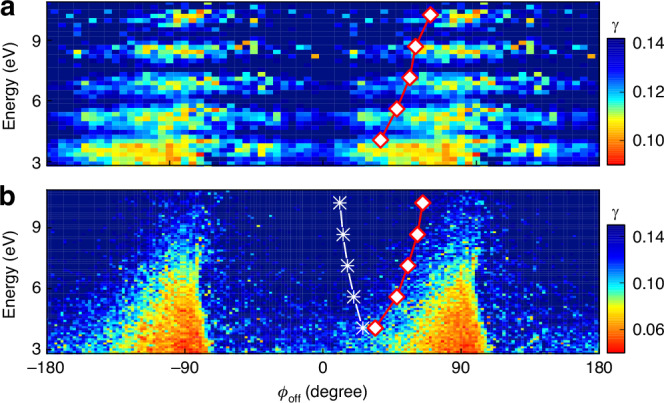
HWHM distribution of the transverse momentum. **a** Measured and (**b**) simulated HWHM distribution of transverse electron momentum, whose value reflects the Coulomb focusing effect. The white diamonds, same to those in Fig. 1, denote the measured or simulated MPEAs of photoelectrons for each ATI ring. The white stars represent the simulated streaking angle of the final momentum of the electron emitted at the instant of the electric field peak for each ATI ring

Essentially, the final momentum of electron can be expressed as^38^:

1 $${\bf{p}}={{\bf{p}}}_{0}({t}_{r})+e{\bf{A}}({t}_{r})+{{\bf{p}}}_{C}(r)$$

where **p**_0_(*t*_*r*_), **A**(*t*_*r*_) and *e* are the initial momentum and the laser vector potential at the time of ionization *t*_*r*_ as well as electron charge, respectively. **p**_*C*_(*r*) is the accumulated momentum drift caused by the position-related Coulomb interaction in the continuum. When the Coulomb interaction is neglected, *t*_*r*_ is the only argument that determines the final momentum of photoelectron. Therefore, there is a one-to-one correspondence between the instant of maximum ionization and the final momentum^4,22,23^, with only those electrons emitted at the instant of the electric field peak contributing to the MPEA in the final PMD. When the Coulomb interaction is taken into account, **p**_*C*_(*r*) is approximately determined by the Coulomb potentials at the tunneling exit *V*(**r**_exit_) and at the infinity $V({\bf{r}}\to \infty )\equiv 0$, so that the position of the tunneling exit **r**_exit_ becomes a relevant factor, and *t*_*r*_ is not the only argument any more. Consequently, Coulomb interaction can not only change the final momentum of each electron trajectory^39^, but also focus a number of electron trajectories with different initial ionization times and tunneling exit positions into the same final momentum as described by Eq. (1) (as shown in Fig. 3b–d). In this case, the overall probability would be the coherent superposition of the weights of all involved electron trajectories, and it depends not only on the weight of each electron trajectory, but also on how many trajectories can be focused together. Therefore, the MPEA and the ionization peak at the tunneling exit do not necessarily tie up. We trace the final momentum of the electron trajectory which is launched at the instant of the electric field peak (see the white stars in Fig. 1c). It is clear that the electrons contributing to the MPEAs are not those emitted at the instant of the electric field peak (see Figs. 1c and 4b). It should be noted that the Coulomb field on single trajectory is included, but the Coulomb focusing is absent in this SCT simulation. In the absence of Coulomb focusing, the MPEA induced by the Coulomb field decreases with increasing energy in the SCT simulation, which contradicts the trend in the experiment and ICCSFA simulation.

In comparison, the adiabatic tunneling exit position *r*_exit_ depends solely on the magnitude of the instantaneous laser electric field at ionization time *t*_*r*_. The tunneling exit in the adiabatic model (tunnel ionization in parabolic coordinates with induced dipole and Stark shift) is given by:

2 $${r}_{{\rm{exit}}}[{\bf{E}}({t}_{r})]=\frac{{I}_{p}+\sqrt{{I}_{p}^{2}-(4-\sqrt{8{I}_{p}})|{\bf{E}}({t}_{r})|}}{2|{\bf{E}}({t}_{r})|}$$

where *I*_*p*_ is the ionization potential of atom. It means that *t*_*r*_ is still the only argument even if the Coulomb interaction is considered in Eq. (1). Therefore, the Coulomb focusing is absent in this adiabatic tunneling model, which explains why the model also predicted an opposite trend to the result of previous experiment and TDSE simulation^21^.

A direct evidence of the Coulomb focusing is the transverse photoelectron momentum distribution along the laser propagation direction *x* and perpendicular to the polarization plane. In this direction, the Coulomb interaction can be readily detected due to excluding the interplay with the laser field. Following the seminal work on Coulomb focusing in tunneling ionization^40^, we utilize a Lorentz distribution to fit the transverse momentum distribution in each bin of the polarization plane (p_y_, p_z_):

3 $$\frac{{\rm{d}}N({{\rm{p}}}_{y},{{\rm{p}}}_{z})}{{{\rm{p}}}_{x}}=\frac{A}{\pi \gamma (1+{{\rm{p}}}_{x}^{2}/{\gamma }^{2})}$$

where p_*x*_, p_*y*_ and p_*z*_ represent the photoelectron momentum along the laser propagation direction, major axis and minor axis of polarization ellipse, respectively. The fit parameter $\gamma =\gamma ({{\rm{p}}}_{y},{{\rm{p}}}_{z})$ corresponds to the half width at half maximum (HWHM). In Fig. 4a, b, we plot the HWHM $\gamma ({{\rm{p}}}_{y},{{\rm{p}}}_{z})$ distributions as a function of photoelectron energy and streaking angle ${\phi }_{{\rm{off}}}$ in the polarization plane with the measurement and semiclassical statistical analysis of ICCSFA, respectively. Here, in order to eliminate the influence of the low ionization yield at the valley of ATI structure, a few-cycle laser pulse is employed to show the energy- and angle-dependent trend of HWHM in the ICCSFA calculation.

**Fig. 5:**
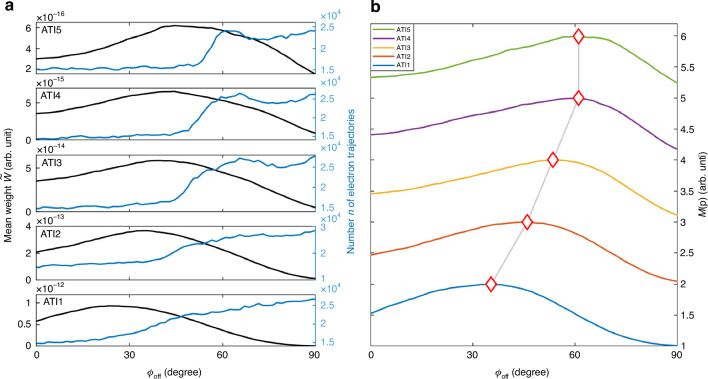
Relation among the probability of photoelectron, the weight and the number of trajectories. **a** The simulated streaking-angle-dependent distributions of the mean weight $\tilde{W}$ (black curve) and number *n* (blue curve) of electron trajectories for different energies. **b** The simulated normalized probability *M*(**p**) involving the combined effect of weight and number of trajectories of photoelectrons for different streaking angles. The white diamonds denote the MPEAs of photoelectron for different ATIs

To get an insight into the Coulomb focusing in angular streaking, we present in Fig. 4 both the measured and simulated MPEAs for each ATI (indicated by the white diamonds in Fig. 4a, b) and the streaking angles of electrons emitted at the instant of the electric field peak (denoted by the white stars in Fig. 4b). The ICCSFA simulation well reproduces the experimentally measured HWHM distribution. Both the measured and simulated results demonstrate that the transverse momentum becomes narrower as the streaking angle ${\phi }_{{\rm{off}}}$ increases (refer to ${0}^{\circ }\, < \,{\phi }_{{{off}}}\, < \,{90}^{\circ }$ and $-{180}^{\circ } \,< \,{\phi }_{{\rm{off}}}\, <\, -{90}^{\circ }$, where the color denotes the value of HWHM), which corresponds to stronger Coulomb focusing with the increase of ${\phi }_{{\rm{off}}}$. The same trend can be found for the MPEAs in the polarization plane (see the white diamonds in Fig. 4a, b). This reveals that, in addition to focusing the transverse momentum, the Coulomb field also focuses electrons into the MPEAs of PMD in the polarization plane. Stronger Coulomb focusing results in more electron trajectories converging into the same momentum bin. This relevance is verified by comparing the HWHMs of the transverse momentum (Fig. 4) with the numbers of trajectories in each momentum bin (the blue lines in Fig. 5a) in the polarization plane. The Coulomb focusing on electrons emitted at the instant of the electric field peak is weak (see the white stars in Fig. 4b). Despite the large weights of these electron trajectories, only a small fraction of them converge and do not contribute to the MPEA with maximum yield due to the weak Coulomb focusing.

The impact of Coulomb focusing on the energy-dependent trend of the MPEAs can be assessed by exploring the relation among the probability of photoelectron, the weight and the number of trajectories. The probability of each momentum bin can be expressed as $M({\bf{p}})={\sum }_{j=1}^{n}{W}_{j}$, where ${W}_{j}$ and *n* are the weight of the *j*th electron trajectory and the number of trajectories in this bin, respectively. We define a mean weight $\tilde{W}$, which allows us to approximate the probability as the product of $\tilde{W}$ and *n*, i.e., $M({\bf{p}})\simeq \tilde{W}\cdot n$. In Fig. 5, we plot the number of trajectories, the mean weight and the probability in each momentum bin for the five ATI rings in the polarized plane. The mean weight $\tilde{W}$ initially increases to the maximum and then decreases with the streaking angle ${\phi }_{{\rm{off}}}$ (indicated by the black lines in Fig. 5a), while the number of trajectories *n* which characterizes the Coulomb focusing, increases with ${\phi }_{{\rm{off}}}$ (represented by the blue lines in Fig. 5a). And the maximum values of $\tilde{W}$ and *n* increase with both the energy and the streaking angle ${\phi }_{{\rm{off}}}$. Consequently, the maximum probability, i.e., the maximum value of the product $M({\bf{p}})\simeq \tilde{W}\cdot n$, increases with both the energy and ${\phi }_{{\rm{off}}}$, which establishes that Coulomb focusing serves as the underlying physical reason responsible for the nonintuitive energy-dependent trend of the streaking angles observed in the previous nonadiabatic tunneling experiment^21^.

## Discussions

**Fig. 6:**
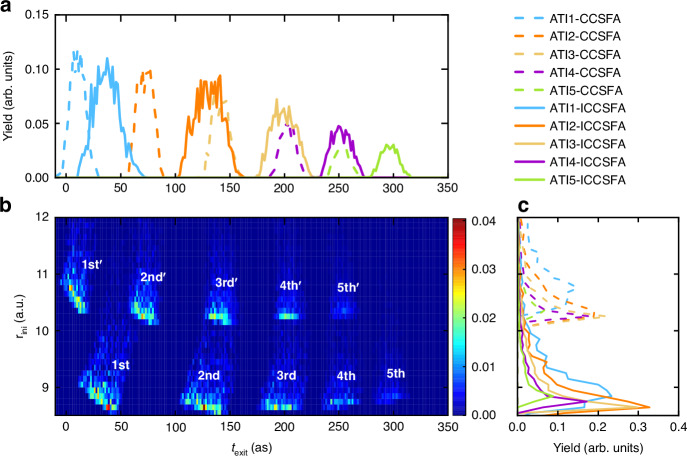
Effect of sub-barrier Coulomb attraction in nonadiabatic tunneling. **a** Simulated birth time tagged ionization rate and initial position of MPEAs for the 1st to 5th ATI rings. **b** The ionization rate integrated over the emission time of the photoelectron. **c** The distribution of the emission time integrated over the initial tunneling position of the photoelectron

In Figs. 1 and 2, we demonstrate that the quantitative agreement between measurements and semiclassical simulations can be achieved only if the ICCSFA incorporates the sub-barrier Coulomb interaction. The sub-barrier Coulomb interaction is the Coulomb attraction on the electron in classically forbidden region. Essentially, it is a process of energy exchange between the electron and its parent ion during the nonadiabatic tunneling^27^. In Fig. 6, we trace back the initial position *r*_ini_ and emission time *t*_exit_ distribution of the electrons contributing to the MPEAs in PMD by artificially enabling (solid lines, simulated with ICCSFA method) or disabling (dashed lines, simulated with CCSFA method) the sub-barrier Coulomb interaction. It reveals that the sub-barrier Coulomb attraction brings electrons closer to the ion (see Fig. 6a, c), which sequentially induces a stronger Coulomb focusing and a broader distribution of initial times (see the comparison between the solid and dashed lines in Fig. 6b and the comparison of HWHM between ICCSFA and CCSFA in Fig. S3 of Supplementary Material). In a word, electrons emitted over a broad time span converge into the MPEAs in angular streaking measurement due to the Coulomb focusing induced by the sub-barrier Coulomb attraction during the nonadiabatic tunneling.

To well account the Coulomb focusing in angular streaking measurement, we establish that the ICCSFA is the most appropriate method relative to CCSFA or SFA, and the reason is summarized as follows. In the SFA model, the influence of the Coulomb potential throughout the entire tunneling ionization process is neglected, and the electron motion is solely driven by the electric field, thereby precluding the Coulomb focusing effect. In the CCSFA model, the Coulomb interaction in the continuum state is considered, leading to the emergence of Coulomb focusing. However, the Coulomb interaction under the barrier is disregarded, severely distorting Coulomb focusing. In this case, the quantitative difference between measurement and simulation using CCSFA can be clearly seen in Fig. 2. By contrast, the ICCSFA model introduces the Coulomb potential not only in the continuum but also under the barrier, resulting in distinct initial conditions of trajectories. Consequently, a more pronounced Coulomb focusing can be accounted, leading to the quantitative agreement between measurement and simulations.

In summary, our jointed experimental and theoretical results have demonstrated the essential role of the Coulomb focusing in angular streaking measurements on laser-induced nonadiabatic tunneling. We have revealed that electrons emitted at different initial times form the MPEAs with maximum yield as a result of Coulomb focusing. The Coulomb focusing triggered by nonadiabatic tunneling disrupts the one-to-one correspondence between the MPEA and ionization burst at the tunneling exit. Our results hold promise for an intuitive interpretation of measured signals in the angular streaking experiments and sheds new light on decoding intricate tunneling dynamics under the classically forbidden barrier in complex with attosecond temporal resolution^41,42^.

## Materials and methods

### Experimental details

Laser pulses were generated by a Spectra-Physics Solstice Ace Ti:Sapphire laser system at a central wavelength of 800 nm. The duration of the laser pulse was 40 fs, and the repetition rate was 1 kHz. Elliptically polarized laser pulses with an ellipticity of 0.79 were produced with the combination of a half-wave plate (HWP) and quarter-wave plate (QWP). The laser pulses were focused into a vacuum chamber by a mirror with a focusing length of 75 mm. A supersonic beam of xenon (seeded in helium) was introduced into the chamber and interacted with the focused laser beams. The energy-resolved angular streaking spectra of photoelectrons from xenon were measured using the COLTRIMS setup (see Fig. 1a)^24,25^. The charged particles were accelerated by the combination of a homogeneous electric field (5.3 V cm^−1^) and a magnetic field (7 Gauss), and their time and position could be recorded by micro-channel plates and delay-line detectors. The count rate of photoelectrons was kept lower than 0.2 per pulse to suppress the false coincidence ratio. The peak laser intensity can be calibrated by measuring the ionization yield ratio of Xe^2+^/Xe^+^ and the ponderomotive potential shift of the ATI peak, which was (45 ± 2) TW cm^−2^ in the measurements^26^.

### Semiclassical simulation and statistical analysis of electron trajectories

Theoretically, we study the Coulomb focusing inside angular streaking technique by performing ICCSFA simulations based on Feynman path integral and semiclassical statistical analysis of electron trajectories. The Coulomb interactions not only in the continuum but also under the classically forbidden barrier are integrated into the ICCSFA method adopted in this work. To solve the saddle-point equation with sub-barrier Coulomb correction, we divide the integral of election action into two parts in the complex-time plane. One of the integral paths is along the imaginary time axis and is strongly correlated with the ionization probability, describing the quantum path evolution under the barrier. The other integration path is along real time axis and describes the propagation of electrons under the laser and Coulomb fields, accompanied by the emission of photoelectrons. Once the quantum trajectory reaches the real time axis, it means that the electron has exited the barrier and entered the classically allowed region. Solving the saddle point equation allows us to obtain the initial distribution of an electron emerging at the tunneling exit, including the initial velocity, position, and the weight of each electron trajectory. Subsequently, the motion of the post-tunneling electron in the combined laser and Coulomb fields is determined by Newton’s equations. The quantum mechanics of electron wavepacket evolution is investigated through a comprehensive statistical analysis of numerous trajectories.

## Supplementary information

Supplementary Information for Coulomb Focusing in Attosecond Angular Streaking

## Data Availability

All data are available from the corresponding authors upon reasonable request.

## References

[1] Hentschel, M. et al. Attosecond metrology. *Nature* **414**, 509–513 (2001). 11734845 10.1038/35107000

[2] Remetter, T. et al. Attosecond electron wave packet interferometry. *Nat. Phys.* **2**, 323–326 (2006). 10.1038/nphys290

[3] Paul, P. M. et al. Observation of a train of attosecond pulses from high harmonic generation. *Science* **292**, 1689–1692 (2001). 11387467 10.1126/science.1059413

[4] Eckle, P. et al. Attosecond angular streaking. *Nat. Phys.* **4**, 565–570 (2008). 10.1038/nphys982

[5] Eckle, P. et al. Attosecond ionization and tunneling delay time measurements in helium. *Science* **322**, 1525–1529 (2008). 19056981 10.1126/science.1163439

[6] Han, M. et al. Complete characterization of sub-coulomb-barrier tunnelling with phase-of-phase attoclock. *Nat. Photonics* **15**, 765–771 (2021). 10.1038/s41566-021-00842-7

[7] Ueda, K. & Ishikawa, K. L. Attoclocks play devil’s advocate. *Nat. Phys.* **7**, 371–372 (2011). 10.1038/nphys1985

[8] Yu, M. et al. Full experimental determination of tunneling time with attosecond-scale streaking method. *Light Sci. Appl.* **11**, 215 (2022). 35798716 10.1038/s41377-022-00911-8 PMC9262890

[9] Nisoli, M. The birth of attochemistry. *Opt. Photonics N.* **30**, 32–39 (2019). 10.1364/OPN.30.7.000032

[10] Krausz, F. & Stockman, M. I. Attosecond metrology: from electron capture to future signalprocessing. *Nat. Photonics* **8**, 205–213 (2014). 10.1038/nphoton.2014.28

[11] Krausz, F. & Ivanov, M. Attosecond physics. *Rev. Mod. Phys.* **81**, 163–234 (2009). 10.1103/RevModPhys.81.163

[12] He, P. L., Ruiz, C. & He, F. Carrier-envelope-phase characterization for an isolated attosecond pulse by angular streaking. *Phys. Rev. Lett.* **116**, 203601 (2016). 27258867 10.1103/PhysRevLett.116.203601

[13] Sainadh, U. S. et al. Attosecond angular streaking and tunnelling time in atomic hydrogen. *Nature* **568**, 75–77 (2019). 30886392 10.1038/s41586-019-1028-3

[14] Camus, N. et al. Experimental evidence for quantum tunneling time. *Phys. Rev. Lett.* **119**, 023201 (2017). 28753333 10.1103/PhysRevLett.119.023201

[15] Landsman, A. S. et al. Ultrafast resolution of tunneling delay time. *Optica* **1**, 343–349 (2014). 10.1364/OPTICA.1.000343

[16] Torlina, L. et al. Interpreting attoclock measurements of tunnelling times. *Nat. Phys.* **11**, 503–508 (2015). 10.1038/nphys3340

[17] Zimmermann, T. et al. Tunneling time and weak measurement in strong field ionization. *Phys. Rev. Lett.* **116**, 233603 (2016). 27341232 10.1103/PhysRevLett.116.233603

[18] Klaiber, M., Hatsagortsyan, K. Z. & Keitel, C. H. Tunneling dynamics in multiphoton ionization and attoclock calibration. *Phys. Rev. Lett.* **114**, 083001 (2015). 25768761 10.1103/PhysRevLett.114.083001

[19] Hofmann, C., Landsman, A. S. & Keller, U. Attoclock revisited on electron tunnelling time. *J. Mod. Opt.* **66**, 1052–1070 (2019). 10.1080/09500340.2019.1596325

[20] Han, M. et al. Unifying tunneling pictures of strong-field ionization with an improved attoclock. *Phys. Rev. Lett.* **123**, 073201 (2019). 31491089 10.1103/PhysRevLett.123.073201

[21] Trabert, D. et al. Nonadiabatic strong field ionization of atomic hydrogen. *Phys. Rev. Lett.* **127**, 273201 (2021). 35061406 10.1103/PhysRevLett.127.273201

[22] Pfeiffer, A. N. et al. Attoclock reveals natural coordinates of the laser-induced tunnelling current flow in atoms. *Nat. Phys.* **8**, 76–80 (2012). 10.1038/nphys2125

[23] Pfeiffer, A. N. et al. Probing the longitudinal momentum spread of the electron wave packet at the tunnel exit. *Phys. Rev. Lett.* **109**, 083002 (2012). 23002743 10.1103/PhysRevLett.109.083002

[24] Dörner, R. et al. Cold target recoil ion momentum spectroscopy: a ‘momentum microscope’ to view atomic collision dynamics. *Phys. Rep.* **330**, 95–192 (2000). 10.1016/S0370-1573(99)00109-X

[25] Ullrich, J. et al. Recoil-ion and electron momentum spectroscopy: reaction-microscopes. *Rep. Prog. Phys.* **66**, 1463–1545 (2003). 10.1088/0034-4885/66/9/203

[26] Wang, C. C. et al. Accurate in situ measurement of ellipticity based on subcycle ionization dynamics. *Phys. Rev. Lett.* **122**, 013203 (2019). 31012706 10.1103/PhysRevLett.122.013203

[27] Yudin, G. L. & Ivanov, M. Y. Nonadiabatic tunnel ionization: looking inside a laser cycle. *Phys. Rev. A* **64**, 013409 (2001). 10.1103/PhysRevA.64.013409

[28] Boge, R. et al. Probing nonadiabatic effects in strong-field tunnel ionization. *Phys. Rev. Lett.* **111**, 103003 (2013). 25166662 10.1103/PhysRevLett.111.103003

[29] Mosert, V. & Bauer, D. Photoelectron spectra with Q_PROP_ and t-SURFF. *Comput. Phys. Commun.* **207**, 452–463 (2016). 10.1016/j.cpc.2016.06.015

[30] Tulsky, V. & Bauer, D. Q_PROP_ with faster calculation of photoelectron spectra. *Comput. Phys. Commun.* **251**, 107098 (2020). 10.1016/j.cpc.2019.107098

[31] Feynman, R. P. Space-time approach to non-relativistic quantum mechanics. *Rev. Mod. Phys.* **20**, 367–387 (1948). 10.1103/RevModPhys.20.367

[32] Feynman, R. P. & Hibbs, A. R. *Quantum Mechanics and Path Integrals* (Dover Publications, Inc., 2005).

[33] Gong, X. C. et al. Energy-resolved ultrashort delays of photoelectron emission clocked by orthogonal two-color laser fields. *Phys. Rev. Lett.* **118**, 143203 (2017). 28430519 10.1103/PhysRevLett.118.143203

[34] Song, X. H. et al. Attosecond time delay of retrapped resonant ionization. *Phys. Rev. Lett.* **121**, 103201 (2018). 30240251 10.1103/PhysRevLett.121.103201

[35] Tong, J. H. et al. Probing resonant photoionization time delay by self-referenced molecular attoclock. *Phys. Rev. Lett.* **129**, 173201 (2022). 36332237 10.1103/PhysRevLett.129.173201

[36] Liu, X. W. et al. Deep learning for Feynman’s path integral in strong-field time-dependent dynamics. *Phys. Rev. Lett.* **124**, 113202 (2020). 32242706 10.1103/PhysRevLett.124.113202

[37] Zhu, M. et al. Tunnelling of electrons via the neighboring atom. *Light Sci. Appl.* **13**, 18 (2024). 38228578 10.1038/s41377-023-01373-2 PMC10791752

[38] Liu, K. L. et al. Detecting and characterizing the nonadiabaticity of laser-induced quantum tunneling. *Phys. Rev. Lett.* **122**, 053202 (2019). 30822014 10.1103/PhysRevLett.122.053202

[39] Pfeiffer, A. N. et al. Recent attoclock measurements of strong field ionization. *Chem. Phys.* **414**, 84–91 (2013). 10.1016/j.chemphys.2012.02.005

[40] Shafir, D. et al. Trajectory-resolved coulomb focusing in tunnel ionization of atoms with intense, elliptically polarized laser pulses. *Phys. Rev. Lett.* **111**, 023005 (2013). 23889394 10.1103/PhysRevLett.111.023005

[41] Huppert, M. et al. Attosecond delays in molecular photoionization. *Phys. Rev. Lett.* **117**, 093001 (2016). 27610849 10.1103/PhysRevLett.117.093001

[42] Jordan, I. et al. Attosecond spectroscopy of liquid water. *Science* **369**, 974–979 (2020). 32820124 10.1126/science.abb0979

