# Supplementary material for: Coulomb focusing in attosecond angular streaking
Source: Light Sci Appl. 2024 Sep 11;13:250. doi: 10.1038/s41377-024-01600-4 (PMC11391041; doi:10.1038/s41377-024-01600-4)
Supplement: Supplementary file 1 — Supplementary Information for Coulomb Focusing in Attosecond Angular Streaking [file 41377_2024_1600_MOESM1_ESM.pdf]

Supplementary Material for "Coulomb Focusing in Attosecond Angular Streaking"

Xiaokai Li, Xiwang Liu, Chuncheng Wang, Shuai Ben, Shengpeng Zhou, Yizhang Yang, Xiaohong Song, Jing Chen, Weifeng Yang & Dajun Ding

1 Experimental results for different atoms

To verify the same energy-dependent trend in different atoms, another measurement for argon (Ar), krypton (Kr) and xenon(Xe) was conducted with the same laser condition. In experiment, the gas mixture of Ar, Kr and Xe is injected into the reaction microscope spectrometer, which ensures the measured ATI spectra originated from the same laser condition (800 nm, ε = 0.84, 86 TW cm^{-2}) for different atoms. The momentum-coincident measurement of parent ion and electron enables the separation of electrons from different target atoms. The measured ATI-resolved offset angles for Ar and Kr are presented in Fig. S1. All of them exhibit the increasing most probable emission angles with energy, indicating a consistent energy-dependent trend observed in the recent measurement on hydrogen [1].

Photoelectron momenta and angular offsets at various laser intensities were also measured, as presented in Fig. S2. The results show that the offset angles are smaller when employing a higher laser intensity which is close to the adiabatic tunneling regime. Therefore, a relatively low laser intensity should be selected for the energy-resolved angular streaking measurements on the nonadiabatic tunneling.

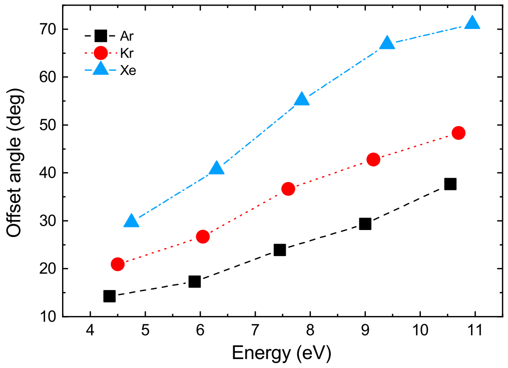

FIG. S1. The measured offset angle as a function of photoelectron energy for Ar, Kr and Xe atoms with the same experiment condition (800 nm, $\varepsilon = 0.84$, 86 TW cm^{-2}).

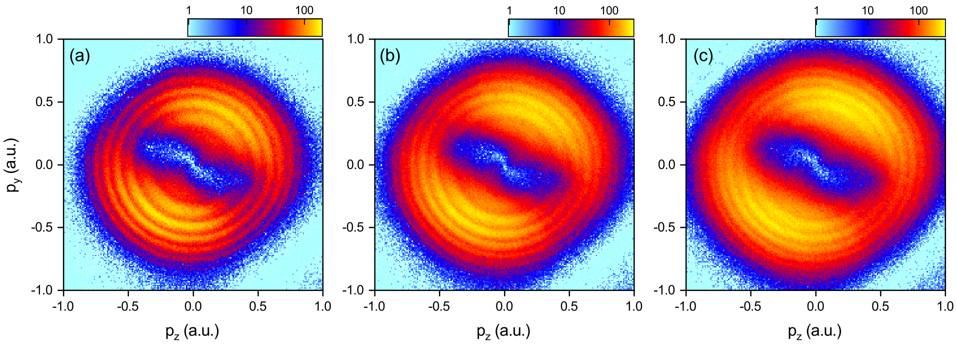

FIG. S2. Measured intensity dependent photoelectron momentum distribution of Xe atom in elliptically polarized laser fields (800 nm, $\varepsilon = 0.84$) with intensity of (a) 56 TW cm^{-2}, (b) 66 TW cm^{-2} and (c) 83 TW cm^{-2}. It shows that the angular offsets decrease with the increased intensity.

2 Improved Coulomb-corrected strong-field approximation (ICCSFA).

In strong field ionization, the transition matrix element is given by

$$M\left(\mathbf{p}\right)=-i\int_{t_0}^{\infty}d\tau\left\langle\Psi_f|\mathbf{r}\cdot\mathbf{E}(\tau)|\psi_0\right\rangle e^{-iS(\mathbf{p},\tau)}, \tag{S1}$$

the action S is a multi-dimensional complex-valued function which is comprised of three components

$$S\left(\mathbf{p},t\right)=S_{\mathrm{V}}\left(\mathbf{p},t\right)+S_{\mathrm{C}}\left(\mathbf{p},t\right)-I_p t. \tag{S2}$$

Here the first component is the action accumulated by the electron in the laser field

$$S_{\mathrm{V}}\left(\mathbf{p},t\right)=\frac{1}{2}\int_{t}^{\infty}d\tau[\mathbf{p}+\mathbf{A}(\tau)]^2, \tag{S3}$$

and the second one is the action accumulated due to the electron with the parent ion

$$S_{\mathrm{C}}\left(\mathbf{p},t\right)=\frac{1}{2}\int_{t}^{\infty}d\tau V[\mathbf{r}(\mathbf{p},\tau)], \tag{S4}$$

where $V[\mathbf{r}(\mathbf{p},\tau)]$ is the ion potential evaluated along the electron trajectory

$$\mathbf{r}(\mathbf{p},\tau)=\int_{t}^{\tau}d\tau'\left(\mathbf{p}+\mathbf{A}\left(\tau'\right)\right). \tag{S5}$$

The third term is related to the evolution of the initial bound state whose energy is -I_p. The saddle point equation [2] follows from Eq.(S2) that

$$\left.\frac{\partial S(\mathbf{p},t)}{\partial t}\right|_{t=t_s}=\left.\frac{\partial S_{\mathrm{V}}(\mathbf{p},t)}{\partial t}\right|_{t=t_s}+\left.\frac{\partial S_{\mathrm{C}}(\mathbf{p},t)}{\partial t}\right|_{t=t_s}-I_p=0. \tag{S6}$$

Here the solution of the saddle point equation is a complex time $t_s=t_r+i\cdot t_i$. The real part t_r of the complex time denotes the initial ionization time when an electron reaches the tunneling exit

and enters the continuum. The imaginary part t_i is closely related to the weight of electron along the quantum path.

The difficulty of solving the accurate saddle point equation (S6) lies in that the potential interaction term S_{C} is not an analytical function of the complex time t. As a result, in the plain SFA and CCSFA methods, this term S_{C} is usually neglected [3–6]. Without the potential interaction, the reduced saddle point equation is

$$\left.\frac{\partial S_{\mathrm{V}}(\mathbf{p},t)}{\partial t}\right|_{t=t_s} - I_p = 0, \tag{S7}$$

which can be easily solved. However, as we have mentioned in the main text, the potential interaction is important to the tunneling. In our work, we provide a self-consistent method to solve Eq. (S6), which includes the sub-barrier potential interaction during the tunneling.

Since that the integrand of S_{V} is an analytic function of complex time t, see Eq. (S3), we can easily get

$$\left.\frac{\partial S_{\mathrm{V}}(\mathbf{p},t)}{\partial t}\right|_{t=t_s} = -\frac{1}{2}[\mathbf{p}+\mathbf{A}(t)]^2. \tag{S8}$$

Solving $\left.\frac{\partial S_{\mathrm{C}}(\mathbf{p},t)}{\partial t}\right|_{t=t_s}$ is a challenge because that $V[\mathbf{r}(\mathbf{p},t)] = Z/\mathbf{r}(\mathbf{p},t)$, with Z the effective charge of the ionic core, is not an analytic function in multidimensional complex plane especially when $\mathbf{r}$ is close to zero. Thus, $\left.\frac{\partial S_{\mathrm{C}}(\mathbf{p},t)}{\partial t}\right|_{t=t_s}$ cannot be directly deduced. To solve this problem, we divide the integral of Eq. (S4) into two parts in complex-time plane

$$\begin{aligned} S_{\mathrm{C}}(\mathbf{p},t) &= \int_t^{\infty} d\tau V[\mathbf{r}(\mathbf{p},\tau)] \\ &= \int_{\mathrm{I}_1} d\tau V[\mathbf{r}(\mathbf{p},\tau)] + \int_{\mathrm{I}_2} d\tau V[\mathbf{r}(\mathbf{p},\tau)], \end{aligned} \tag{S9}$$

where the integral-path I_1 is along the imaginary time from $t = t_s = t_r + i \cdot t_i$ to $t = t_r$, i.e., changing the imaginary part from t_i to 0 while keeping the real part t_r unchanged, which describes the path evolution under the barrier, i.e., the sub-barrier dynamics. The integral-path I_2 is along real time from $t = t_r$ to ∞, i.e., the real-time part. Then the potential interaction term in the saddle point equation would be

$$\left.\frac{\partial S_{\mathrm{C}}(\mathbf{p},t)}{\partial t}\right|_{t=t_s} = \left.\left[\frac{\partial}{\partial t}\int_{\mathrm{I}_1} d\tau V[\mathbf{r}(\mathbf{p},\tau)] + \frac{\partial}{\partial t}\int_{\mathrm{I}_2} d\tau V[\mathbf{r}(\mathbf{p},\tau)]\right]\right|_{t=t_s}, \tag{S10}$$

where the first term can be written as

$$\begin{aligned}\left.\frac{\partial}{\partial t}\int_{\mathrm{I}_1} d\tau V[\mathbf{r}(\mathbf{p},\tau)]\right|_{t=t_s} &= \lim_{\Delta t\to 0}\frac{\int_{t_i+\Delta t}^{0} V\left[\mathbf{r}\left(\mathbf{p},t_r+i\cdot\tau\right)\right]d\tau - \int_{t_i}^{0} V\left[\mathbf{r}\left(\mathbf{p},t_r+i\cdot\tau\right)\right]d\tau}{\Delta t} \\ &= -V\left[\mathbf{r}\left(\mathbf{p},t_s\right)\right],\end{aligned} \tag{S11}$$

and the second term can be written as

$$\begin{aligned}\left.\frac{\partial}{\partial t}\int_{\mathrm{I}_2} d\tau V[\mathbf{r}(\mathbf{p},\tau)]\right|_{t=t_s} &= \lim_{\Delta t\to 0}\frac{\int_{t_r+\Delta t}^{\infty} V[\mathbf{r}(\mathbf{p},\tau)]d\tau - \int_{t_r}^{\infty} V[\mathbf{r}(\mathbf{p},\tau)]d\tau}{\Delta t} \\ &= -V\left[\mathbf{r}\left(\mathbf{p},t_r\right)\right].\end{aligned} \tag{S12}$$

Substituting Eqs. (S8)-(S12) to Eq. (S6), then we can get the saddle point equation with the potential interaction which can be solved numerically

$$\frac{1}{2}\left(\mathbf{p}+\mathbf{A}\left(t_s\right)\right)^2 + V\left[\mathbf{r}\left(t_s\right)\right] = -I_p - V\left[\mathbf{r}\left(t_r\right)\right]. \tag{S13}$$

Here, $\mathbf{r}$ is the position of the electron, $V[\mathbf{r}((t_s))] = -Z_{eff}/|\mathbf{r}(t_s)|$ is the potential energy at the tunneling entrance and $V[\mathbf{r}((t_r))] = -Z_{eff}/|\mathbf{r}(t_r)|$ is the potential energy at the tunneling exit, where $Z_{eff} = \sqrt{2I_p}$ is the effective charge of the ionic core.

Physically, the saddle point equation Eq. (S13) describes energy conservation during the tunneling. Without the Coulomb correction, i.e., within SFA, the energy was assumed to change

from the ground state with energy $-I_p$ before tunneling to the continuum state with zero energy after tunneling. The electron could not experience the effect of the ionic Coulomb potential during the tunneling. However, actually, the electron is not totally free during the tunneling, but still experiences the attraction force of parent ion under the classically forbidden barrier owing to the Coulomb potential. As a result, the variation of the potential energy before and after tunneling should be equal to $V\left[\mathbf{r}\left(t_r\right)\right]+I_p$. Therefore, Eq. (S13) describes the energy conservation when the sub-barrier Coulomb interaction and potential energy are included.

After the tunneling, the subsequent motion of the electron in the continuum is governed by the combined laser and Coulomb fields. The initial distribution for an electron emerging at the tunneling exit, including the initial velocity, position, and the weight of each electron trajectory can be obtained from the saddle point equation (S13). Then, the motion of the electron in the real-time propagation is determined by Newton's equations:

$$\begin{cases} \dot{\mathbf{p}}(t) = -\nabla_{\mathbf{r}} V[\mathbf{r}(t)] - \mathbf{E}(t) \\ \\ \dot{\mathbf{r}}(t) = \mathbf{p}(t) \end{cases} . \tag{S14}$$

In this work, we employed a fourth-order Runge-Kutta method to solve Eq. (S14). The phase in real time is

$$S\left(\mathbf{p}, t_r\right) = \int_{t_r}^{\infty} \left(\frac{1}{2}\mathbf{v}^2(\tau) - \frac{Z_{eff}}{|\mathbf{r}(\tau)|} + I_p\right) d\tau, \tag{S15}$$

where $\mathbf{v}(\tau)$ is the instantaneous velocity of the electron. As the Coulomb interactions both during the tunneling and in the continuum have been included, we name this method improved Coulomb-

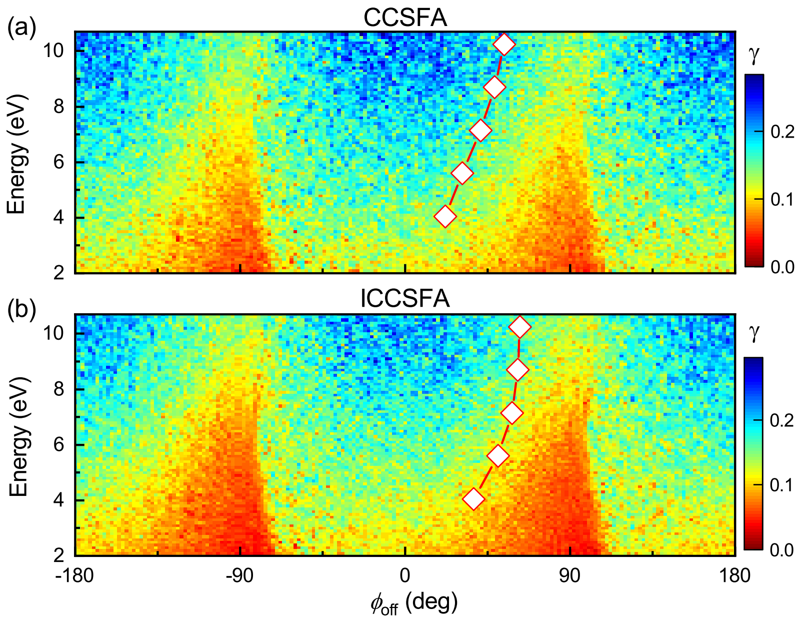

FIG. S3. Simulated HWHM distributions of the photoelectron transverse momentum from (a) CCSFA and (b) ICCSFA. The red diamond-shaped curves denote the simulated MPEAs of photoelectrons with different energies.

corrected strong-field approximation (ICCSFA).

Figure S3 shows the simulated HWHM distributions of transverse momentum and MPEAs with CCSFA and ICCSFA methods. The HWHM values around MPEAs in ICCSFA simulation are relatively smaller than those in CCSFA calculation, which means a stronger Coulomb focusing and induces the increase of MPEAs.

References

1. D. Trabert *et al.*, Nonadiabatic strong field ionization of atomic hydrogen, Phys. Rev. Lett. **127**, 273201 (2021).
2. L. Torlina, F. Morales, J. Kaushal, I. Ivanov, A. Kheifets, A. Zielinski, A. Scrinzi, H. G. Muller, S. Sukiasyan, M. Ivanov, and O. Smirnova, Interpreting attoclock measurements of tunnelling times, Nat. Phys. **11**, 503 (2015).
3. L. V. Keldysh, Ionization in the field of a strong electromagnetic wave, Sov. Phys. JETP **20**, 1307 (1965).
4. F. H. M. Faisal, Multiple absorption of laser photons by atoms, J. Phys. B **6**, L89 (1973).
5. H. R. Reiss, Effect of an intense electromagnetic field on a weakly bound system, Phys. Rev. A **22**, 1786 (1980).
6. M. Lewenstein, P. Balcou, M. Y. Ivanov, A. L'Huillier, and P. B. Corkum, Theory of high-harmonic generation by low-frequency laser fields, Phys. Rev. A **49**, 2117 (1994).
